# Metagenomes of tropical soil-derived anaerobic switchgrass-adapted consortia with and without iron

**DOI:** 10.4056/sigs.3377516

**Published:** 2013-02-25

**Authors:** Kristen M. DeAngelis, Patrik D’Haeseleer, Dylan Chivian, Blake Simmons, Adam P. Arkin, Konstantinos Mavromatis, Stephanie Malfatti, Susannah Tringe, Terry C. Hazen

**Affiliations:** 1 Microbiology Department, University of Massachusetts, Amherst MA USA; 2 Microbial Communities Group, Deconstruction Division, Joint BioEnergy Institute, Emeryville CA USA; 3 Lawrence Livermore National Laboratory, Livermore CA USA; 4 Physical Biosciences Division, Lawrence Berkeley National Laboratory, Berkeley CA USA; 5 Technologies Division, Joint BioEnergy Institute, Emeryville CA USA; 6 Sandia National Lab, Livermore CA USA; 7 Department of Energy Joint Genome Institute, Walnut Creek CA USA; 8 Ecology Department, Earth Sciences Division, Lawrence Berkeley National Laboratory, Berkeley, California, USA; 9 Department of Civil and Environmental Engineering, University of Tennessee, Knoxville, Tennessee, USA; 10 Biosciences Division, Oak Ridge National Laboratory, Oak Ridge, Tennessee, USA

**Keywords:** Anaerobic decomposition, switchgrass, *Panicum virgatum*, tropical forest soil, feedstock-adapted consortia, bacteria, archaea, metagenomics

## Abstract

Tropical forest soils decompose litter rapidly with frequent episodes of anoxia, making it likely that bacteria using alternate terminal electron acceptors (TEAs) such as iron play a large role in supporting decomposition under these conditions. The prevalence of many types of metabolism in litter deconstruction makes these soils useful templates for improving biofuel production. To investigate how iron availability affects decomposition, we cultivated feedstock-adapted consortia (FACs) derived from iron-rich tropical forest soils accustomed to experiencing frequent episodes of anaerobic conditions and frequently fluctuating redox. One consortium was propagated under fermenting conditions, with switchgrass as the sole carbon source in minimal media (SG only FACs), and the other consortium was treated the same way but received poorly crystalline iron as an additional terminal electron acceptor (SG + Fe FACs). We sequenced the metagenomes of both consortia to a depth of about 150 Mb each, resulting in a coverage of 26× for the more diverse SG + Fe FACs, and 81× for the relatively less diverse SG only FACs. Both consortia were able to quickly grow on switchgrass, and the iron-amended consortium exhibited significantly higher microbial diversity than the unamended consortium. We found evidence of higher stress in the unamended FACs and increased sugar transport and utilization in the iron-amended FACs. This work provides metagenomic evidence that supplementation of alternative TEAs may improve feedstock deconstruction in biofuel production.

## Introduction

Development of renewable, sustainable biofuels from plant feedstock material has emerged as a key goal of the US Department of Energy. The use of lignocellulose as a renewable energy source has many advantages, above all that lignocellulose is the most abundant biopolymer on earth, with its production independent of food agriculture [1]. The deconstruction of plant biomass is a key first step in the conversion of plant sugars to biofuels, though this step has posed a great challenge to making biofuels economically viable. The major hurdles involve both lignin occlusion of cellulose and lignin derivatives that inhibit lignocellulose deconstruction and fuel synthesis [1]. Lignin is also a potentially valuable waste stream that is currently burned to produce energy as heat [2]. Part of the impact of this work is the discovery of enzymes and pathways in natural ecosystems that function to liberate lignin from cellulose. These discoveries promise to both provide insight into the natural processes of plant lignin decomposition, as well as improve efficiency of biofuels production.

The microbial communities present in the wet tropical soils of Puerto Rican rain forests are promising in providing pathways to overcome the challenges of lignocellulose deconstruction. These tropical soil communities are responsible for near complete decomposition of leaf plant litter in as little as eighteen months [3], which is interesting considering that the soils experience strong fluctuations in redox potential, switching from a completely oxic state to an anoxic state on a daily or weekly basis [4,5]. We have also observed considerable microbial activity and plant litter decomposition under anaerobic conditions in the lab and field [6-9]. This is at odds with the current paradigm of the “enzyme latch hypothesis,” which posits that oxidative enzyme activities are the rate-limiting steps of plant litter decomposition [10-12]. Understanding the enzymes employed by native tropical soil microbes to deconstruct lignocellulose has the potential to illuminate the mechanisms of fast anaerobic lignocellulose decomposition.

## Classification and features

Two metagenomes were generated from feedstock-adapted consortia, originally derived from tropical forest soil communities [Table 1]. Soil samples were collected from a wet subtropical lower montane forest in the Luquillo Experimental Forest, which is part of the NSF-sponsored Long-Term Ecological Research program in Puerto Rico (18°18′N, 65°50′W). The fieldwork was conducted and samples collected and transported under USDA permit number P526P-08-00634. Soils are acidic (pH 5.5), clayey ultisols with high iron and aluminum content and characterized by a fluctuating redox that ranges from oxic to anoxic on a timescale of weeks [4,5,15]. Soils were collected from the Bisley watershed, 250 meters above sea level (masl) from the 0–10 cm depth, using a 2.5-cm diameter soil corer. Cores were stored intact in Ziploc bags at ambient temperature and immediately transported to the lab, where they were used for growth inoculum.

**Table 1:** Classification and general features of the four metagenome data sets according to the Minimum Information about Genomes and Metagenomes (MIMS) standards [13].

**MIMS ID**	**Property**	**Term**	**Evidence code**^a^
	Current classification	Metagenome ecological metagenome terrestrial metagenome	TAS [5]
	Carbon source	Switchgrass	IDA
	Energy source	Switchgrass	IDA
	Terminal electron receptor	Iron reduction or fermentation	TAS [8]
MIGS-6	Habitat	Consortia (mixed community) derived from wet tropical forest soils	TAS [8]
MIGS-14	Pathogenicity	none	NAS
MIGS-4	Geographic location	Wet tropical forest, Puerto Rico, USA	
MIGS-5	Sample collection time	April, 2009	
MIGS-4.1	Latitude	18°18′N	
MIGS-4.2	Longitude	65°50′W	
MIGS-4.3	Depth	0-10 cm	TAS [8]
MIGS-4.4	Altitude	250 masl	TAS [8]

^a^ Evidence codes - IDA: Inferred from Direct Assay; TAS: Traceable Author Statement (i.e., a direct report exists in the literature); NAS: Non-traceable Author Statement (i.e., not directly observed for the living, isolated sample, but based on a generally accepted property for the species, or anecdotal evidence). These evidence codes are from the Gene Ontology project [14].

For adaptation to growth on feed-stocks as sole carbon source, tropical forest soils were homogenized then used to inoculate basal salts minimal medium (BMM) [16] containing trace minerals [17,18], vitamins [19], and buffered to pH 5.5 to match the measured soil pH using MES. Soils were added at a rate of 0.5 g (wet weight) per 200 mL BMM, and the resulting mixture was incubated anaerobically at ambient temperatures for 8 weeks with 10 g L^-1^ dried, ground switchgrass as the sole carbon source. Samples of switchgrass (MPV 2 cultivar) were kindly provided by the laboratory of Dr. Ken Vogel (USDA, ARS, Lincoln, NE). Soluble iron was added to a final concentration of 5 mM. A stock solution of soluble iron was obtained by adding ferric chloride hexahydrate [Fe(III)] to a solution of nitrilotriacetic acid disodium salt and sodium bicarbonate. Dinitrogen gas was bubbled through media to remove any dissolved O_2_, and containers were quickly sealed with airtight stoppers to maintain anaerobic conditions. Containers were autoclaved for 20 min at 121°C. Anaerobic switchgrass-adapted consortia were enriched from tropical forest soils by passaging the communities two times for ten weeks each, with switchgrass as the sole carbon source, under anaerobic conditions with and without supplemental iron.

## Metagenome sequencing information

### Metagenome project history

These metagenomes were selected based on the ability of the consortia to mineralize switchgrass as the sole C source anaerobically, and represented two distinct metabolisms for deconstructing switchgrass that are both likely to be prevalent under natural field conditions. Sequence analysis of the small subunit ribosomal RNA genes revealed that growth on switchgrass as the sole carbon source resulted in a richness of 84 taxa, while inclusion of iron in the consortia growth media resulted in a richness of 336 taxa; this was in comparison to the richness of the original soil sample which was 1,339 taxa [20] based on 97% identity.

### Growth conditions and DNA isolation

Consortia were grown for metagenomic DNA sequencing in the same manner as described for the cultivation of communities, as above. DNA was extracted using a CTAB extraction method, which is the standard operating procedure recommended by the Joint Genome Institute. Cells from the consortia were pelleted by centrifugation and reconstituted in TE to an equivalent OD (600 nm) of about 1.0 using direct counts. Lysozyme was added (final concentration 1.3 mg per ml) and incubated for 5 minutes at room temperature, then 10% SDS (33 µl per ml) and proteinase K (final concentration 5.5µl per ml) was added and incubated at 37^o^C for 1 hour. Sodium chloride (5M stock added to final concentration 0.22 M) was added, then the CTAB/NaCl buffer was added both at 0.075 ml per ml starting volume. This mix was incubated at 65^o^C for 10 minutes. Chloroform:isoamyl alcohol (24:1) was added at 0.2 vol, then centrifuged at 14,000 x *g* for 10 minutes at room temperature. DNA in the aqueous phase was extracted again with phenol:chloroform:isoamyl alcohol (25:24:1), subjected to an ethanol precipitation, and the DNA pellet finally reconstituted at 37^o^C for 20 minutes in TE plus RNAse. The quantity and quality of the extraction were checked by gel electrophoresis using JGI standards.

### Metagenome sequencing and assembly

The metagenomes were sequenced using the Illumina GaII sequencing platform. Two types of short-insert (300 bp) paired-end libraries were generated, with and without PCR amplification after adapter ligation. All general aspects of the library construction process can be accessed via the DOE Joint Genome Institute website [21]. 16.2 Gb of Illumina GaII sequence data were generated for the PR soil-derived Feedstock-adapted consortia SG + Fe sample and 33.9 Gb Illumina GaII sequence data were generated for the PR soil-derived Feedstock-adapted consortia SG only sample. Raw Illumina metagenomic reads were trimmed using a minimum quality score cutoff of 10. Trimmed, paired-end Illumina reads were assembled using SOAPdenovo v1.05 [22] with a range of Kmers (85, 89, 93, 97, 101, 105). Default settings for all SOAPdenovo assemblies were used (flags: –d 1 and –R). Contigs generated by each assembly (6 total contig sets) were sorted into two pools based on length. Contigs smaller than 1,800 bp were assembled using Newbler (Life Technologies, Carlsbad, CA) in an attempt to generate larger contigs (flags: -tr, -rip, -mi 98, -ml 80). All assembled contigs larger than 1,800 bp, as well as the contigs generated from the final Newbler run, were combined using minimus 2 (flags: -D MINID=98 -D OVERLAP=80)(AMOS [23]). The assembly was a result of two rounds of sequencing in this manner, with and without amplification. Table 2 presents the project information and its association with MIGS version 2.0 compliance [13]. These sequences are currently available to the public at IMG/M.

**Table 2:** Project information

**MIGS ID**	**Property**	**Term**
MIGS-31	Finishing quality	Standard Draft
MIGS-28	Libraries used	Illumina standard paired-end library (0.3 kb insert size)
MIGS-29	Sequencing platforms	Illumina GaIIX
MIGS-31.2	Fold coverage	26.1466 × (PR soil-derived FAC SG + Fe) 81.1761 × (PR soil-derived FAC SG only)
MIGS-30	Assemblers	SOAPdenovo v1.05, Newbler v2.5, minimus2
MIGS-32	Gene calling method	Glimmer
	GOLD ID	Gm00278
	IMG Project ID	18182
	Project relevance	biotechnological

### Metagenome annotation

Prior to annotation, all sequences were trimmed to remove low quality regions falling below a minimum quality of Q13, and stretches of undetermined sequences at the ends of contigs are removed. Low complexity regions are masked using the dust algorithm from the NCBI toolkit and very similar sequences (similarity > 95%) with identical 5’ pentanucleotides are replaced by one representative, typically the longest, using uclust [24]. The gene prediction pipeline included the detection of non-coding RNA genes (tRNA, and rRNA) and CRISPRs, followed by prediction of protein coding genes.

Identification of tRNAs was performed using tRNAScan-SE-1.23 [25]. In case of conflicting predictions, the best scoring predictions were selected. Since the program cannot detect fragmented tRNAs at the end of the sequences, we also checked the last 70 nt of the sequences by comparing these to a database of nt sequences of tRNAs identified in the isolate genomes using blastn [26]. Hits with high similarity were kept; all other parameters are set to default values. Ribosomal RNA genes (tsu, ssu, lsu) were predicted using the hmmsearch [27] with internally developed models for the three types of RNAs for the domains of life. Identification of CRISPR elements was performed using the programs CRT [28] and PILERCR [29]. The predictions from both programs were concatenated and, in case of overlapping predictions, the shorter prediction was removed.

Identification of protein-coding genes was performed using four different gene calling tools, GeneMark (v.2.6r) [29] or Metagene (v. Aug08) [30], prodigal [31] and FragGenescan [32] all of which are *ab initio* gene prediction programs. We typically followed a majority rule based decision scheme to select the gene calls. When there was a tie, we selected genes based on an order of gene callers determined by runs on simulated metagenomic datasets (Genemark > Prodigal > Metagene > FragGeneScan). At the last step, CDS and other feature predictions were consolidated. The regions identified previously as RNA genes and CRISPRs were preferred over protein-coding genes. Functional prediction followed and involved comparison of predicted protein sequences to the public IMG database using the usearch algorithm [24], the COG db using the NCBI developed PSSMs [33], the pfam db [34] using hmmsearch. Assignment to KEGG Ortholog protein families was performed using the algorithm described in [35].

## Metagenome properties

The metagenomes were sequenced at a total size of 152,660,070 bp for the SG only FACS and 154,120,208 bp for the SG + Fe FACS. The GC content of these metagenomes was 41.18% for SG only and 46.02% for SG + Fe FACs. This sequencing included 197,271 and 193,491 predicted genes with 98.85% and 99.62% predicted protein-coding genes for SG only and SG + Fe FACs, respectively. A total of 127,406 and 129,389 of the protein coding genes, or 64.58% and 66.87% of the total predicted protein-coding genes, were assigned to a putative function with the remaining annotated as hypothetical proteins for SG only and SG + Fe FACs, respectively.

The iron-amended consortia (SG + Fe) was significantly enriched in protein-coding genes with putative function for 11 of the 25 general COG categories that genes were assigned. The largest differences were observed for genes associated with amino acid transport and metabolism (E), carbohydrate transport and metabolism (G), and secondary metabolites biosynthesis, transport, and catabolism (Q), which were all enriched in the iron-amended compared to the unamended FACs. Genes assigned to translation, ribosomal structure and biogenesis (J) and transcription (K) were significantly depleted in the iron-amended compared to the unamended consortia. The properties and the statistics of the genome are summarized in Table 3, Table 4 and Table 5.

**Table 3:** Summary of metagenomes

**Metagenome**	**Size (Mb)**	**# scaffolds**	**GOLD ID**	**GOLD sample idID**
SG only	152.66	57,147	Gm00278	Gs0000888
SG + Fe	154.12	65,160	Gm00278	Gs0000889

**Table 4:** Nucleotide content and gene count levels of the metagenomes

	**SG only Metagenome**		**SG + Fe Metagenome**	
	Number	% of Total	Number	% of Total
DNA, total number of bases	152,660,070	100.00%	154,120,208	100.00%
DNA coding number of bases	130,438,005	85.44%	136,080,382	88.29%
DNA G+C number of bases	62,858,797	41.18%*	70,930,796	46.02%*
DNA scaffolds	57,147	100.00%	65,160	100.00%
CRISPR Count	51		4	
Genes total number	197,271	100.00%	193,491	100.00%
Protein coding genes	195,006	98.85%	192,751	99.62%
RNA genes	2,265	1.15%	740	0.38%
rRNA genes	294	0.15%	16	0.01%
5S rRNA	106	0.05%	9	0.00%
16S rRNA	75	0.04%	3	0.00%
18S rRNA	1	0.00%	4	0.00%
tRNA genes	1,971	1.00%	724	0.37%
Protein coding genes with function prediction	127,406	64.58%	129,389	66.87%
without function prediction	67,600	34.27%	63,362	32.75%
not connected to SwissProt Protein Product	195,006	98.85%	192,751	99.62%
Protein coding genes with enzymes	33,383	16.92%	30,632	15.83%
w/o enzymes but with candidate KO based enzymes	26,793	13.58%	32,919	17.01%
Protein coding genes connected to KEGG pathways3	37,533	19.03%	34,348	17.75%
not connected to KEGG pathways	157,473	79.83%	15,8403	81.87%
Protein coding genes connected to KEGG Orthology (KO)	63,949	32.42%	57,111	29.52%
not connected to KEGG Orthology (KO)	131,057	66.44%	135,640	70.10%
Protein coding genes connected to MetaCyc pathways	32,243	16.34%	29,552	15.27%
not connected to MetaCyc pathways	162,763	82.51%	163,199	84.34%
Protein coding genes with COGs3	121,020	61.35%	123,077	63.61%
with Pfam3	115,645	58.62%	118,589	61.29%
with TIGRfam3	33,743	17.10%	33,969	17.56%
in internal clusters	77,655	39.36%	73,856	38.17%
Protein coding genes coding signal peptides	48,556	24.61%	49,644	25.66%
Protein coding genes coding transmembrane proteins	43,693	22.15%	43,726	22.60%
COG clusters	4,125	84.65%	3,974	81.55%
KOG clusters	0	0.00%	0	0.00%
Pfam clusters	4,447	37.33%	4,293	36.04%
TIGRfam clusters	2,580	64.13%	2,489	61.87%

* GC percentage shown as count of G's and C's divided by a total number of G's, C's, A's, and T's. This is not necessarily synonymous with the total number of bases.

**Table 5:** Number of genes associated with the 25 general COG functional categories.

ID	Name	SG only	SG + Fe	R	P-value
J	Translation, ribosomal structure and biogenesis	6,659	6,055	4.57	*** 0.000
A	RNA processing and modification	21	22	0	*n.s.*
K	RNA processing and modification	21	22	0	*n.s.*
L	Replication, recombination and repair	6,248	6,103	1.36	0.086
B	Chromatin structure and dynamics	49	35	0.21	*n.s.*
D	Cell cycle control, cell division, chromosome partitioning	1,457	1,68	1.35	0.089
Y	Nuclear structure	-	-	-	-
V	Defense mechanisms	2,884	3,232	-2.11	* 0.018
T	Signal transduction mechanisms	10,430	10,271	2.01	* 0.022
M	Cell wall/membrane/envelope biogenesis	8,396	8,753	-1.67	* 0.047
N	Cell motility	3,150	3,146	-1.2	*n.s.*
Z	Cytoskeleton	39	43	-0.29	*n.s.*
W	Extracellular structures	2	1	0	*n.s.*
U	Intracellular trafficking, secretion, and vesicular transport	2,438	2,525	-0.56	*n.s.*
O	Posttranslational modification, protein turnover, chaperones	3,893	3,914	0.14	*n.s.*
C	Energy production and conversion	8,221	8,426	0.08	*n.s.*
G	Carbohydrate transport and metabolism	13,038	14,361	-3.69	*** 0.000
E	Amino acid transport and metabolism	9,571	10,682	-5.33	*** 0.000
F	Nucleotide transport and metabolism	2,808	3,022	-2.21	* 0.014
H	Coenzyme transport and metabolism	5,193	5,080	2.17	* 0.015
I	Lipid transport and metabolism	3,034	3,375	-1.9	* 0.029
P	Inorganic ion transport and metabolism	5,914	6,171	0.12	*n.s.*
Q	Secondary metabolites biosynthesis, transport and catabolism	1,608	1,916	-2.51	** 0.006
R	General function prediction only	15,442	15,796	-0.87	*n.s.*
S	Function unknown	10,667	10,106	0.6	*n.s.*

^a^P-value symbols denote * P<0.05, ** P<0.01, *** P<0.001, and *n.s.* indicates not significant.

## Taxonomic diversity

The taxonomic diversity and phylogenetic structure of the two metagenomes was determined based on all genes, classifying at a minimum 60% identity to members of the listed phyla. The phylogeny reported is the one in use in IMG/M [36], which uses the phylogeny described as part of the genomic encyclopedia of *Bacteria* and *Archaea* (GEBA) project [37].

Both consortia were dominated by representative genes belonging to the *Firmicutes*, which accounted for 20 and 23% of the counts in the SG only and SG + Fe FACs, respectively. In terms of relative abundance, the next most dominant genes belonged to the phylum *Bacteroidetes*, accounting for 7% of the counts, and *Proteobacteria*, accounting for 6% of the counts. Members of the archaeal phylum *Euryarchaeota* accounted for 2.6% and 1.34% of the SG only and SG + Fe FACs gene counts, respectively. There were very few documented members of the *Eukaryota*, accounting for less than one-tenth of one percent. Plasmid population-associated genes were dominated by those associated with *Firmicutes* and *Proteobacteria*, and these were outnumbered by double-stranded DNA viruses by about two to one.

**Figure 1:**
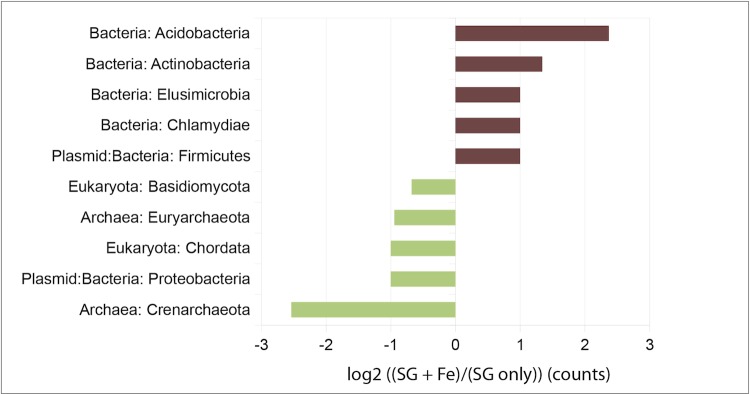
Phyla that are at least 2-fold differentially represented in one metagenome compared to the other, and had greater than one representative detected. Phyla with gene counts over-represented in the iron-amended consortium (SG + Fe) are colored brown, while phyla with gene counts over-represented in the unamended consortium (SG only) are colored light green.

Differences were observed in abundance of genes in many phyla, which was expected given the much higher richness observed by pyrosequencing the small subunit ribosomal RNA genes in consortia amended with iron as terminal electron acceptor. To visualize which phyla were over- or under-represented in gene counts for each metagenome, we chose to present the phyla that were at least two-fold (double) differentially represented in the iron-amended consortia compared to the unamended consortia. Fold-differences were calculated by dividing the counts detected in SG+Fe FACs divided by counts detected in SG only FACs, and the log2 of fold differences are presented in Figure 1. Phyla that were over-represented in the iron-amended FACs included *Acidobacteria* (5.17-fold enriched), *Actinobacteria* (2.5-fold), *Elusimicrobia* (2-fold), and *Chlamydiae* (2-fold), which are all *Bacteria*; also over-represented in the SG + Fe were *Firmicutes* plasmids (2-fold). Of these, only the *Acidobacteria* and *Actinobacteria* were abundant (>2% relative abundance), with the rest detected in the tens of counts per metagenome. Phyla that were under-represented in the iron-amended FACs compared to the SG only FACs included *Basidiomycota* (domain *Eukaryota*, 0.625-fold), *Euryarchaeota* (domain *Archaea*, 0.52-fold), *Chordata* (domain *Eukaryota*, 0.5-fold), *Proteobacteria* Plasmids (0.5-fold), and *Crenarchaeota* (domain *Archaea*, 0.17-fold) [Table 6].

**Table 6:** Overview of taxonomic diversity in metagenomes.

Domain	Phylum	SG only Count	%	SG + Fe Count	%
*Archaea*					
	*Crenarchaeota*	575	0.29	88	0.05
	*Euryarchaeota*	5,089	2.58	2,590	1.34
	*Nanoarchaeota*	1	0	0	0
*Bacteria*					
	*Acidobacteria*	478	0.24	2,392	1.24
	*Actinobacteria*	1,113	0.56	2,756	1.42
	*Aquificae*	100	0.05	99	0.05
	*Bacteroidetes*	14,680	7.44	14,937	7.72
	*Chlamydiae*	16	0.01	35	0.02
	*Chlorobi*	460	0.23	530	0.27
	*Chloroflexi*	1,073	0.54	2042	1.06
	*Chrysiogenetes*	13	0.01	18	0.01
	*Cyanobacteria*	742	0.38	823	0.43
	*Deferribacteres*	117	0.06	121	0.06
	*Deinococcus-Thermus*	158	0.08	275	0.14
	*Dictyoglomi*	132	0.07	144	0.07
	*Elusimicrobia*	17	0.01	35	0.02
	*Fibrobacteres*	30	0.02	36	0.02
	*Firmicutes*	38,958	19.75	44,858	23.18
	*Fusobacteria*	533	0.27	534	0.28
	*Gemmatimonadetes*	11	0.01	29	0.01
	*Lentisphaerae*	113	0.06	165	0.09
	*Nitrospirae*	70	0.04	80	0.04
	*Planctomycetes*	375	0.19	448	0.23
	*Proteobacteria*	11,289	5.72	11,803	6.1
	*Spirochaetes*	2,460	1.25	3,795	1.96
	*Synergistetes*	314	0.16	460	0.24
	*Tenericutes*	30	0.02	34	0.02
	*Thermodesulfobacteria*	36	0.02	43	0.02
	*Thermotogae*	330	0.17	414	0.21
	*Verrucomicrobia*	450	0.23	611	0.32
*Eukaryota*					
	*Apicomplexa*	25	0.01	19	0.01
	*Arthropoda*	32	0.02	30	0.02
	*Ascomycota*	64	0.03	59	0.03
	*Bacillariophyta*	7	0	8	0
	*Basidiomycota*	8	0	5	0
	*Chlorophyta*	5	0	5	0
	*Chordata*	32	0.02	29	0.01
	*Microsporidia*	1	0	0	0
	*Nematoda*	7	0	3	0
	*Streptophyta*	73	0.04	66	0.03
Plasmid:Archaea					
	*Crenarchaeota*	5	0	2	0
	*Euryarchaeota*	4	0	4	0
Plasmid:Bacteria					
	*Actinobacteria*	3	0	3	0
	*Bacteroidetes*	2	0	1	0
	*Firmicutes*	23	0.01	32	0.02
	*Proteobacteria*	32	0.02	29	0.01
Viruses					
	ds DNA viruses, no RNA stage	113	0.06	97	0.05
	ss DNA viruses	1	0	0	0

While gene counts of representative phyla suggest phylogenetic differences, these data are certainly biased towards phyla that have more sequenced representatives. Additionally, phyla that are included in coverage of popular universal small subunit rRNA primers are also may be over-represented in these analyses because of their over-representation in the databases. While the relative abundances of between-phyla comparisons may be questionable based on differential representation in the database, the relative abundances of taxa within a phyla is reflective of the distinct metabolic conditions afforded by growth of consortia with lignocellulose as sole C source either with or without iron as an additional terminal electron acceptor.

In an additional, separate experiment, we tested the effects of additional terminal electron acceptors on the ability of feedstock-adapted consortia to degrade switchgrass, which included iron as well as sulfate and nitrate, with switchgrass-only as a control. In this additional experiment, we analyzed the resulting microbial communities by the taxonomic marker 16S ribosomal RNA gene sequence libraries [8]. These communities were grown from the SG only FACs whose metagenomic sequences are presented here. Further passages were made before community analysis, making these consortia from this additional experiment less rich and characterized by fewer dominant species. Because these communities are simpler, we are able to more closely examine the relationships among taxa and co-occurrences under varying availability of terminal electron acceptors.

We observed some differences in taxon occurrence and functional gene abundance between the iron-amended and iron-unamended metagenomes, and used network analysis to illustrate the phylogenetic basis of taxon co-occurrence among differences in availability of terminal electron acceptors. Network analyses were constructed by calculating all possible correlations between taxa using Pearson's correlation coefficient, then discarding any pairwise correlations that did not meet the criteria for a “connection”, which was a minimum r value of 0.9 and minimum P-value of 0.01. Network analysis was conducted based on the methods presented in Barberán *et al.* [38] in R using the packages igraph [39], Hmisc [40], multtest [41], doMC [42], and foreach [43].

**Figure 2:**
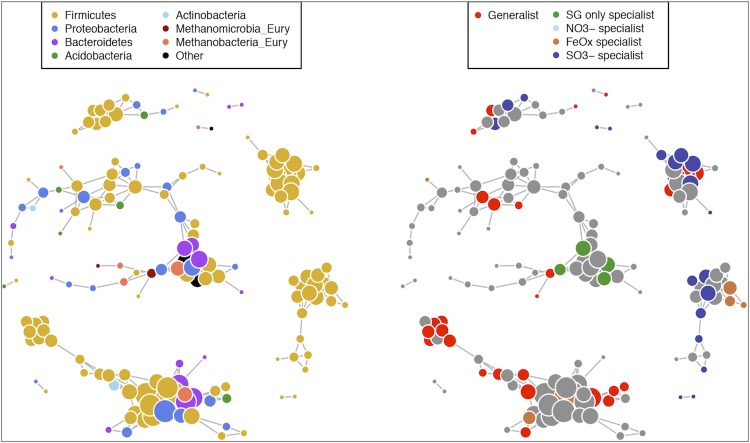
Network analysis of feedstock adapted consortia grown on switchgrass only (SG only), SG plus iron oxides (FeOx), SG plus nitrate (NO_3_^-^), or SG plus sulfate (SO_3_^-^). Each point represents one taxon, and the size of the point corresponds to the number of connections (edges) associated with the taxon. Edges (grey lines) indicate a minimum correlation of Pearson r = 0.9 as well as statistical significance (P<0.01). On the left, taxa are colored by taxonomy according to their assigned phylum; on the right, taxa are colored based on whether they are generalists (present in all four treatments) or specialists (present in one treatment only and absent in the rest).

These networks were strongly dominated by a few taxa, as evidenced by the large number of singletons (661 taxa, 62%) and doubletons (167 taxa, 16%) detected among the total taxa detected (1,060). These singleton and doubleton taxa were not included in the network analysis, leaving 232 taxa for analyzing co-occurrences. Of the 1,060 taxa included in this analysis, 170 taxa met the minimum criteria for a significant connection, with 579 connections between them. Taxa are mapped in Figure 2 with colors corresponding to phylum (left) as well as by generalist or specialist (right), where generalists were defined as taxa detected in all TEA treatments, while specialists were defined as taxa detected in only one treatment.

As expected due to the static anaerobic conditions, networked communities are dominated by *Firmicutes*, which are prevalent in all clusters. *Firmicutes* also dominated the SG only and SG + Fe FACs, accounting for 20 and 23% of total richness, respectively. The *Firmicutes* contain the *Clostridiales*, which are fast-growing obligate anaerobes, fermenters, and well-known lignocellulolytic microbes [44-46]. In our consortia networks, the *Firmicutes* tended to either be generalists or switchgrass-only specialists, which may also explain their prevalence in our metagenomes. The specialists were dominated by *Firmicutes*, with the notable observation that there were no nitrate specialists detected by this method. Of the remaining specialists, there were more sulfate-specialists than any other kind, followed by switchgrass-only specialists, then iron specialists. All iron specialists were *Firmicutes*; this was somewhat surprising considering that the best-known iron reducers are in the phylum *Proteobacteria*, including *Geobacter* and *Shewanella* [47,48]. However, these taxa were notably absent in previous phylogenetic and metagenomic analyses of wet tropical forest soils of Puerto Rico [9,20], and there are actually a wide diversity of iron-reducing bacteria within the *Firmicutes*. In the network, *Firmicutes* also tended to co-occur either with each other, forming large cliques, or with taxa from diverse phyla. Generalists were mostly *Firmicutes*, but also included representatives from the phyla *Proteobacteria* and *Methanomicrobia* (of the Euryarchaeota). These phyla are known to accommodate some well-known K-selected species; taxa that are not fast growers but have persistent growth and are able to survive under a range of conditions [12,49].

## Functional genetic diversity

**Figure 3:**
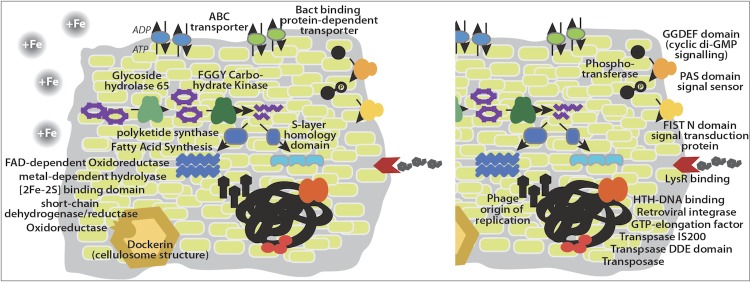
Illustration of pfams that were differentially represented in SG only compared to SG + Fe. On the left, pfams are listed for the consortium grown in switchgrass only with no iron (SG only), and on the right, pfams are listed for the consortium grown in switchgrass with iron (SG + Fe). This illustration is based on data from Table 7.

Analysis of pfams that were significantly different between iron-amended FACs (SG+Fe) and unamended FACs (SG only) suggested that there were strong differences between the function and metabolic status of the two consortia (Table 7, Figure 3). There were 15 pfams that were significantly enriched in the SG + Fe FACs, compared to 23 pfams enriched in SG only compared to SG + Fe. The pfams enriched in the SG + Fe suggested that the addition of iron caused the consortia to be overall more efficient at transporting xylose and other nutrients, as evidenced by the large number of ABC transporters and other bacterial transporter systems. Transporters made up the bulk of the identified pfams, representing the most abundant pfam domains differentially detected in the SG + Fe compared to the SG only FACs. There was also evidence that the increased transport of carbon and other nutrients resulted in increased biosynthesis of biomass and secondary metabolites, with pfams such as S-layer homology domain, oxidoreductase family domains, [Fe-S] binding domains, and polyketide synthesis domains. The SG + Fe FACs were also significantly enriched in glycosyl-hydrolase family 65 domains compared to the switchgrass only FACs, suggesting that this community had more members that were able to utilize the switchgrass for energy and microbial biomass.

**Table 7:** Report of pfams that were significantly enriched^†^

ID	SG only	R	SG + Fe		Description	
Enriched in SG+Fe FACs						
pfam00005	2,409	-7.38	8.10e-14	3,025		ABC transpoerter
pfam00528	1,817	-7.36	9.02e-14	2,348		Bacterial binding protein-dependent transport systems
pfam02653	394	-6.24	2.23e-10	605		Bacterial binding protein-dependent transport systems
pfam00106	420	-4.88	5.38e-07	589		short-chain dehydrogenases/reductases family
pfam01979	163	-5.55	1.44e-08	287		large metal dependent hydrolase superfamily
pfam08352	99	-6.43	6.20e-11	218		C-terminus of oligopeptide ABC transporter ATP binding proteins
pfam02894	146	-4.1	2.03e-05	231		Oxidoreductase family, C-terminal alpha/beta domain
pfam02782	113	-4.33	7.56e-06	193		FGGY carbohydrate kinase family
pfam00395	130	-3.98	3.39e-05	208		S-layer homology domain
pfam01266	93	-3.77	8.17e-05	156		FAD dependent oxidoreductase family
pfam02801	47	-4.13	1.79e-05	99		Beta-ketoacyl-ACP synthase (fatty acid synthesis)
pfam00404	2	-6.02	8.86e-10	43		Dockerin: protein domain in cellulosome cellular structure
pfam01799	24	-3.71	1.02e-04	59		[2Fe-2S] binding domain
pfam03632	19	-3.8	7.32e-05	52		glycoside hydrolase family 65
pfam08659	1	-5.41	3.23e-08	33		polyketide synthase domain, catalyses the first step in the reductive modification of the beta-carbonyl centers in the growing polyketide chain
Enriched in SG only FACs						
pfam00990	697	5.95	1.33e-09	508		GGDEF domain, cyclic di-GMP synthesis involved in intracellular signaling
pfam03466	499	6.29	1.60e-10	330		LysR substrate binding domain, similar to periplasmic binding protein
pfam00126	476	5.71	5.71e-09	326		Helix-turn-helix DNA binding domain
pfam00583	1,048	3.88	5.26e-05	905		Acetyltransferase (or transacetylase)
pfam00989	569	3.89	4.92e-05	459		PAS domain, signal sensor
pfam00563	262	5.43	2.89e-08	157		EAL domain, possible diguanylate phosphodiesterase with metal-binding site
pfam01473	135	8.26	1.11e-16	31		Putative cell wall binding repeat
pfam02311	405	3.78	7.82e-05	314		rabinose-binding and dimerization domain of the AraC regulatory protein
pfam00665	201	4.53	2.94e-06	124		Retroviral integrase
pfam02378	145	4.25	1.08e-05	84		Phosphotransferase system, EIIC, part of a sugar-specific permease system
pfam00801	163	3.71	1.03e-04	106		Polycystic-kidney disease domain, usually involved in mediating protein-protein interactions
pfam01797	119	3.84	6.07e-05	69		Transposase IS200, for transposition of insertion elements
pfam01609	119	3.76	8.44e-05	70		Transposase DDE domain, for transposition of insertion elements
pfam01011	75	4.54	2.83e-06	30		beta propeller, found in several enzymes which utilize pyrrolo-quinoline quinone as a prosthetic group
pfam00367	80	4.35	6.80e-06	35		phosphotransferase system, EIIB
pfam02302	105	3.69	1.13e-04	60		phosphoenolpyruvate: sugar phosphotransferase system (PTS) system, Lactose/Cellobiose specific IIB subunit
pfam09681	47	5.93	1.53e-09	5		N-terminal phage replisome organizer, origin of phage replication
pfam01978	83	3.83	6.47e-05	42		sugar-specific transcriptional regulator of the trehalose/maltose ABC transporter
pfam03143	52	3.75	8.81e-05	21		GTP-binding elongation factor family
pfam09820	35	4.41	5.09e-06	7		predicted AAA-ATPase domain
pfam08350	33	4	3.22e-05	8		domain of unknown function, so far found only at the C-terminus of archaean proteins
pfam08495	36	3.74	9.02e-05	11		FIST N domain: novel sensory domain present in signal transduction proteins
pfam09373	22	4.45	4.34e-06	1		Pseudomurein-binding repeat, pseudomurein being a cell-wall structure
pfam08004	18	3.96	3.71e-05	1		domain of unknown function, so far found only among archaeal proteins

^†^in either the iron-amended consortia (SG + Fe FAC, upper half of table) or the unamended feedstock-adapted consortia (SG only FAC, lower half of table).

In contrast, the pfams detected in the SG only FACs that were significantly enriched compared to the SG + Fe FACs hinted at stressful conditions in survival of the community without the addition of the exogenous terminal electron acceptor iron. There were a number of intracellular signaling domains that were enriched, suggesting that there were more interactions among remaining community members that grow under these conditions. There was also evidence of enrichment for mobile genetic elements and viral DNA transfer, evidenced by increased detection of transposase domains, retroviral integrases, and phage replication domains. It has been demonstrated that communities under stress have higher transfer rate of mobile genetic elements, potentially as a mechanism to induce better survival strategies [50]. These differences in detected pfams at the DNA level suggest that the metagenomic sequencing of the SG only FACs occurred prior to the community adapting to the lack of exogenous terminal electron acceptors. That is, our sequencing was performed before the community had arrived at a new equilibrium, and over the course of selection for anaerobic growth of tropical soil communities on switchgrass as sole C source, the communities were unable to adapt to the lack of iron as terminal electron acceptor.

## Functional Genes Related to Feedstock Deconstruction

To recover genes that were specifically involved in switchgrass deconstruction, we used blastp to pull out sequences from the annotated, assembled metagenomes that had E-value of 1e-20 or better. The target list contained 101 proteins, consisting of glycosyl hydrolases, lignases, and other proposed lignocellulose-degrading enzymes based on genome analysis of the isolate *Enterobacter lignolyticus* SCF1 [51], which originated from these same soils. This resulted in 1,001 hits from both the SG only and SG + Fe FACs, but 54 and 198 targets on scaffolds longer than 10kb from the SG only and SG + Fe FACs, respectively. These results are summarized in Table 8, where we report the number of genes clustered by COG ID number. There were 13 COGs that contain genes detected in both FACs, three COGs with genes detected in the SG only FAC but not the SG + Fe FAC, and 24 COGs with genes detected in the SG + Fe FAC but not the SG only FAC. This imbalance in target lignocellulolytic genes, with many more genes detected with iron amendment than without the TEA amendment, supports our conclusion that iron addition improves lignocellulose decomposition among these FACs.

**Table 8:** Count of genes in COGs that bear protein sequence homology to target lignocellulolytic genes of interest.

**COG ID**	**SG only**	**SG + Fe**	**COG Description**
COG1472	11	23	Beta-glucosidase-related glycosidases
COG3250	11	7	Beta-galactosidase/beta-glucuronidase
COG5001	9	9	Predicted signal transduction protein containing a membrane domain, an EAL and a GGDEF domain
COG1028	4	33	Dehydrogenases with different specificities (related to short-chain alcohol dehydrogenases)
COG0300	3	2	Short-chain dehydrogenases of various substrate specificities
COG4221	3	2	Short-chain alcohol dehydrogenase of unknown specificity
COG1012	2	11	NAD-dependent aldehyde dehydrogenases
COG0677	2	3	UDP-N-acetyl-D-mannosaminuronate dehydrogenase
COG3384	2	3	Uncharacterized conserved protein
COG0280	1	3	Phosphotransacetylase
COG1344	1	3	Flagellin and related hook-associated proteins
COG3325	1	2	Chitinase
COG0277	1	1	FAD/FMN-containing dehydrogenases
COG0179	1		2-keto-4-pentenoate hydratase/2-oxohepta-3-ene-1,7-dioic acid hydratase (catechol pathway)
COG1874	1		Beta-galactosidase
COG2132	1		Putative multicopper oxidases
COG1129		20	ABC-type sugar transport system, ATPase component
COG2723		11	Beta-glucosidase/6-phospho-beta-glucosidase/beta-galactosidase
COG0411		9	ABC-type branched-chain amino acid transport systems, ATPase component
COG0673		8	Predicted dehydrogenases and related proteins
COG0036		4	Pentose-5-phosphate-3-epimerase
COG1455		4	Phosphotransferase system cellobiose-specific component IIC
COG1486		4	Alpha-galactosidases/6-phospho-beta-glucosidases, family 4 of glycosyl hydrolases
COG2200		4	FOG: EAL domain
COG0366		3	Glycosidases
COG1004		3	Predicted UDP-glucose 6-dehydrogenase
COG3842		3	ABC-type spermidine/putrescine transport systems, ATPase components
COG3845		3	ABC-type uncharacterized transport systems, ATPase components
COG0435		2	Predicted glutathione S-transferase
COG0583		2	Transcriptional regulator
COG0812		2	UDP-N-acetylmuramate dehydrogenase
COG3836		2	2,4-dihydroxyhept-2-ene-1,7-dioic acid aldolase
COG4213		2	ABC-type xylose transport system, periplasmic component
COG4214		2	ABC-type xylose transport system, permease component
COG0376		1	Catalase (peroxidase I)
COG0410		1	ABC-type branched-chain amino acid transport systems, ATPase component
COG1640		1	4-alpha-glucanotransferase
COG1921		1	Selenocysteine synthase [seryl-tRNASer selenium transferase]
COG1960		1	Acyl-CoA dehydrogenases
COG2368		1	Aromatic ring hydroxylase
COG2373		1	Large extracellular alpha-helical protein
Total Result	54	**197**	

## Conclusion

Metagenome sequencing of iron-amended and unamended feedstock-adapted consortia suggests that iron amendment results in microbial communities that are more active or more efficient at lignocellulose degradation. This is evidenced by the increased abundance of genes associated carbohydrate transport and decreased abundance of genes associated with cell maintenance and growth. The iron amendment was only applied after one generation of anaerobic growth, so it is possible that further generations of growth in the presence of iron would result in consortia better able to degrade lignocellulosic feedstocks. This research also supports the possibility that anaerobic lignocellulose deconstruction could benefit from metabolism supplemented by additional TEAs.
